# Detailed Characterization of the Cooperative Binding of Piperine with Heat Shock Protein 70 by Molecular Biophysical Approaches

**DOI:** 10.3390/biomedicines8120629

**Published:** 2020-12-18

**Authors:** Gabriel Zazeri, Ana Paula Ribeiro Povinelli, Marcelo de Freitas Lima, Marinônio Lopes Cornélio

**Affiliations:** 1 Departamento de Física, Instituto de Biociências, Letras e Ciências Exatas (IBILCE), UNESP, Rua Cristovão Colombo 2265, CEP 15054-000 São José do Rio Preto, SP, Brazil; gabriel.zazeri@unesp.br (G.Z.); ana.povinelli@unesp.br (A.P.R.P.); 2 Departamento de Química, Instituto de Biociências, Letras e Ciências Exatas (IBILCE), UNESP, Rua Cristovão Colombo 2265, CEP 15054-000 São José do Rio Preto, SP, Brazil; marcelo.f.lima@unesp.br

**Keywords:** heat shock protein 70, Hsp70, piperine, fluorescence spectroscopy, molecular docking, molecular dynamics, molecular biophysics

## Abstract

In this work, for the first time, details of the complex formed by heat shock protein 70 (HSP70) independent nucleotide binding domain (NBD) and piperine were characterized through experimental and computational molecular biophysical methods. Fluorescence spectroscopy results revealed positive cooperativity between the two binding sites. Circular dichroism identified secondary conformational changes. Molecular dynamics along with molecular mechanics Poisson Boltzmann surface area (MM/PBSA) reinforced the positive cooperativity, showing that the affinity of piperine for NBD increased when piperine occupied both binding sites instead of one. The spontaneity of the complexation was demonstrated through the Gibbs free energy (∆G < 0 kJ/mol) for different temperatures obtained experimentally by van’t Hoff analysis and computationally by umbrella sampling with the potential of mean force profile. Furthermore, the mean forces which drove the complexation were disclosed by van’t Hoff and MM/PBSA as being the non-specific interactions. In conclusion, the work revealed characteristics of NBD and piperine interaction, which may support further drug discover studies.

## 1. Introduction

Heat shock proteins (HSPs) constitute the first line of protection for cells exposed to stressful conditions [1]. HSPs belong to the family of intracellular molecular chaperones, which are involved in many cellular processes including protein folding, prevention of protein aggregation, modulation of protein complexes and protein transport between cellular compartments [2].

The comprehension of HSPs functions was very accepted by the scientific community until Asea et al. [3] initiated a paradigm in the understanding of the function of one of the heat shock proteins (HSP70), revealing that this protein may be found in the extracellular medium acting as an inflammatory cytokine that stimulates innate immune response through the activation of Nuclear Factor-κB (NF-κB) [4,5,6], which in turn is responsible for the transcription of more than 150 inflammatory cytokines genes including TNF-α, IL-1β and IL6 [6].

HSP70 is a 70 kDa protein that consists of an independent conserved N-terminal nucleotide binding domain (NBD ≈ 40 kDa) with ATPase activity, a substrate binding domain (SBD ≈ 25 kDa) and a weakly conserved C-terminal domain [2]. The NBD and SBD are linked by a short inter-domain linker [7]. The NBD consists of two subdomains, I and II, which are further divided into regions a and b. The Ia and IIa regions interact with ATP [7]. The interaction with ATP leads the NBD structure to conformational changes that affect the affinity of HSP70 for its receptors (TLRs), revealing the plasticity of this protein [8]. Cheeseman et al. [9] and Jones et al. [2] identified some ligands that caused conformational changes in NBD, reinforcing the plasticity of the domain. Moreover, the authors highlighted the need to find small ligands with the potential to inhibit HSP70. Considering the NBD structural flexibility, the key to inhibit the cytokine function of HSP70 may be the search for small molecules able to induce conformational changes that reduce the affinity for the receptors.

**Figure 1:**
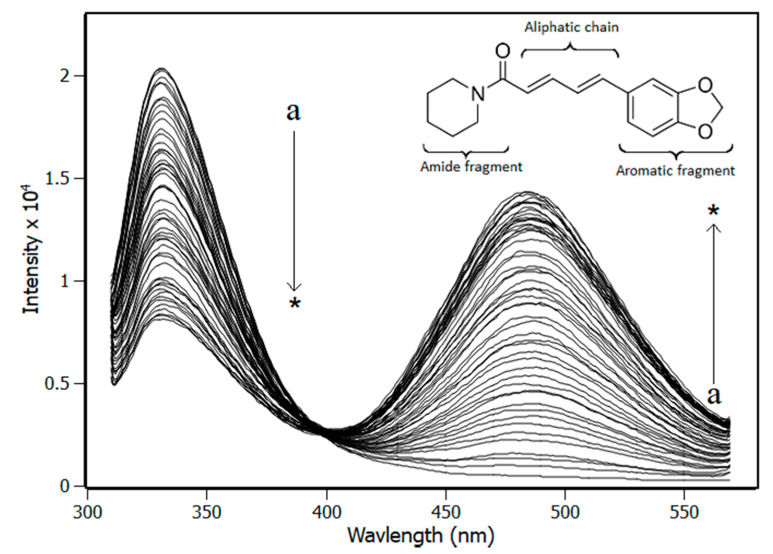
Spectra of fluorescence emission of nucleotide binding domain (NBD) obtained from titration experiments with increments in the concentration of piperine (pH 7.4, T = 288 K, λ_exc_ = 295 nm). (NBD) = 4.0 μM; piperine titrations with increment of 0.5 μM (a → * = 0 μM → 20 μM).

Studies reported in the literature showed by means of biological assays that piperine, a bioactive natural product (inset of Figure 1) [10], inhibited IL-1β-mediated activation of NF-κB, leading to the downregulation of pro-inflammatory proteins in human osteoarthritis chondrocyte [11], in human interleukin 1β-stimulated fibroblast-like synoviocytes and in rat arthritis models [12]. Further, it was reported that piperine also inhibited the LPS-mediated activation of NF-κB in RAW 264.7, not allowing the expression of inflammatory mediators [13]. Although it was recently reported in the literature that HSP70 also triggers the NF-κB activation pathway playing a similar role to IL-1β and LPS, there are no studies either at a cellular level or at a molecular level regarding the interaction of the anti-inflammatory molecule piperine and HSP70.

From this perspective, the present work comes to describe a detailed biophysical characterization of the interaction between NDB and piperine to point out the main features of the interaction, supporting drug discovery teams in order to give them an insight into the possible inhibitory role of piperine. Fluorescence spectroscopy and circular dichroism spectroscopic methods were employed to disclose the number of binding sites, the cooperativity, the binding affinity, the thermodynamic parameters of interaction and the protein conformational changes due to the interactions. To have a complete description of the complex, molecular docking and dynamics were employed to predict and confirm the binding sites, to disclose the molecular interactions involved and to calculate the binding free energy.

## 2. Materials and Methods

### 2.1. Reagents

Piperine (>97%) was purchased from Sigma-Aldrich Chemical Co. (Schnelldorf, Bavaria, Germany), as dibasic sodium phosphate (>99%) reagents, anhydrous citric acid (>99%) and sodium chloride (>99%). Lyophilised NBD (>97%) was purchased from GenScript. Methanol alcohol was purchased from Dynamics Química Contemporânea LTDA (Indaiatuba, SP, Brazil). Ultrapure water was prepared by a Millipore water purification system—Direct-Q UV-3 (Merck KGaA, Darmstadt, Germany). Lyophilized NBD was reconstituted in 50 mM phosphate buffer containing 150 mM of sodium chloride, and the pH was adjusted to 7.4 with anhydrous citric acid. Stock solutions of piperine were prepared in pure methanol. The concentrations of piperine and NBD solutions were determined by UV-VIS experiments performed on a Biospectro spectrophotometer (Biospectro, Curitiba, PR, Brazil), using the extinction coefficient at 16,500 M^−1^cm^−1^ at 345 nm for piperine and 20,525 M^−1^cm^−1^ at 280 nm for NBD.

### 2.2. Steady-State Fluorescence Spectroscopy

Fluorescence experiments were performed on the Lumina (Thermo Fisher Scientific, Waltham, MA, USA) stationary state spectrofluorimeter equipped with a thermal bath and Xenon lamp. A 100 μL quartz cuvette with a 10 × 2 mm optical path was used in the experiments. The widths of the excitation and the emission slits were adjusted to 10 nm. A wavelength of 295 nm was used to excite the single tryptophan residue of NBD (Trp90). The emission spectra were obtained in the range of 305 to 570 nm with a resolution of 1.0 ± 5.0 nm. Each emission point collected was the average of 15 accumulations. The software ScanWave was used to collect the measured data.

In the binding equilibrium experiments, aliquots of piperine (increment of 0.5 μM) were added in NBD solution at 4 μM. Measurements were performed at 288, 298 and 308 K. In the interaction density function analysis, small aliquots of piperine (increments of 1 μM) were added to NBD solutions at 4 μM, 6 μM and 8 μM at a fixed temperature (288 K). In all experiments, the final volume of methanol in the buffer was less than 1.0%.

The correction of the inner filter effects was performed with Equation (1), where *F_corr_* and *F_obs_* are corrected and observed fluorescence intensities, and *A_ex_* and *A_em_* are the absorbance at the excitation and the emission wavelengths, respectively, considering a cuvette of 10 × 10 mm of optical path [14].

(1) $$F_{\mathrm{corr}}={\;F}_{\mathrm{obs}}\cdot \;10^{\frac{\left(5\cdot A_{\mathrm{ex}}+{\;A}_{\mathrm{em}}\right)}{10}}$$

### 2.3. Time-Resolved Fluorescence

Fluorescence lifetime measurements were performed using a mini-tau filter-based fluorescence lifetime spectrometer coupled to a time-correlated single-photon counting (TCSPC) system (Edinburgh Instruments, Livingston, UK). Aliquots of piperine were added in the NBD solution at 4 μM. The piperine concentration varied from 0 to 20 μM. Experiments were carried out at 298 K.

The sample was excited at 295 nm using a picosecond pulsed light emitting diode (LED), and fluorescence decay was collected using a 340 nm filter. The fluorescence decay profile (Figure S1) was fitted using multiexponential decay (Equation (2)), where τ_i_ is the lifetime of each component, and α_i_ is the contribution of each component to total fluorescence decay. The average lifetime <τ_avg_> was calculated using Equation (3) (Table S1).

(2) $$I_{T}=\;\overset{n}{\underset{i=1}{\sum }}\alpha_{i}\cdot e^{\frac{-T}{\tau_{i}}}$$

(3) $${\;\tau }_{\mathrm{avg}}=\frac{\alpha_{1}\tau_{1}{}^{2}+\alpha_{2}\tau_{2}{}^{2}}{\alpha_{1}\tau_{1}+\alpha_{2}\tau_{2}}$$

### 2.4. Circular Dichroism

Circular dichroism spectra were recorded at 288, 298 and 308 K on a Jasco J-815 spectropolarimeter model DRC-H (Jasco, Easton, MD, USA) equipped with a demountable quartz cell with a 0.01 cm optical path length. The CD spectra were recorded from the 200 to 260 nm range with a scan rate of 20 nm/min and a spectral resolution of 0.1 nm. For each spectrum, 15 accumulations were performed. The molar ratios of NBD and piperine were 1:0, 1:2.5, 1:5, 1:7.5, 1:10 and 1:12.5, and the buffer spectrum was subtracted. The ellipticity θ collected in millidegrees was converted to mean residue ellipticity [θ] (deg.cm^2^.dmol^−1^) using Equation (4).

(4) $$\left[\theta \right]=\frac{\theta \left(\mathrm{mdeg}\right)}{10\cdot \left[P\right]\cdot l\cdot n}$$

The secondary structures’ percentages were calculated with CDPro, applying the CONTIN method with the SP43 protein library [15].

### 2.5. Molecular Docking

The piperine structures used in the molecular docking were obtained from ab initio calculations from our previous work [16]. The NBD structure used in the molecular docking was extracted from PDB (1S3X). AutoDockTools [17] software of the MGL program Tools 1.5.4 was used to prepare the NBD by adding polar hydrogen atoms and Gasteiger charges. The maps were generated by the AutoGrid 4.2 program [18] with a spacing of 0.541 Å, a dimension of 126 × 126 × 126 points and grid center coordinates of 51.315, 43.754 and 48.905 for x, y and z coordinates, respectively. The AutoDock 4.2 program [17] was used to investigate the NBD binding sites using the Lamarckian genetic algorithm (LGA) with a population size of 150, a maximum number of generations of 27,000 and energy evaluations equal to 2.5 × 10^6^. The other parameters were selected as software defaults. To generate different conformations, the total number of runs was set to 100. The final conformations were chosen among the most negative energies and belonging to the most representative cluster (Figure S2). The final conformations were visualized on VMD [19]. The binding microenvironment was generated by LigPlot [20].

### 2.6. Molecular Dynamics

The simulations of the complex NBD/piperine were performed with the GROMOS54a6 force field [21] by Gromacs v.5.1.4 [22]. The complex was placed in a rectangular box, solvated with simple point charge water (SPC) [23] and neutralized with NaCl in a concentration of 150 mM. The energy minimization was performed with the steepest descent. The first step of equilibration was performed in an NVT ensemble for 100 ps. The system was coupled to a V-rescale thermostat [24] at 298 K. All bonds were constrained with the LINCS algorithm [25], the cut-off for short-range non-bonded interactions was set at 1.4 nm and long-range electrostatics were calculated using the particle mesh ewald (PME) algorithm [26]. The second step of equilibration was performed in the NPT ensemble coupled to a Parrinello-Rahman barostat [27] to isotropically regulate the pressure for 100 ps. The pulling of piperine from the NBD pocket was performed without restraints to allow the protein conformational changes. The reaction coordinate ξ was chosen as being the distance between the Thr14 oxygen atom (O index 97) and piperine carbon atom (CAA 3802) for piperine into binding site 1 (Figure S3a) and between the Leu309 carbon atom (CA index 3073) and piperine carbon atom (CAL index 3800) for piperine into binding site 2 (Figure S3b). Piperine was pulled away from the NBD binding site in a Z direction until the reaction coordinate reached 7 nm for binding site 1 and 6 nm for binding site 2, using a spring constant of 1000 kJ/mol^−1^nm^−2^ and a pull rate of 0.01 nm/ns (Figure S4). A sampling of the pullings was analyzed to guarantee a good sampling (Figure S5). The potential of mean force (PMF) profile [28] along the reaction coordinate was calculated with the WHAM method [29]. Statistical errors were estimated with a bootstrap analysis, with 1000 bootstraps properly autocorrelated.

The free energy of the binding process of piperine toward NBD was calculated by a G_mmpbsa tool [30], using the molecular mechanics Poisson Boltzmann surface area (MM/PBSA) method applied to the snapshots obtained from the molecular dynamics simulations. The snapshots were extracted from the trajectory after the system reached equilibrium, which was verified by the root mean square deviation (RMSD) obtained by the program *gmx rms* from Gromacs (Figure S6). The snapshots were extracted in intervals of 250 ps. The coarse grid-box (cfac) was set as 2 and the finner grid-box (fadd) was set as 20. The concentration of positive and negative ions was set as 0.150, being the positive and negative radii set as 0.95 and 1.81 Å, which correspond to sodium and chloride atoms, respectively. The values for the vacuum (vdie) and solvent (sdie) dielectric constants were set as 1 and 80, respectively. The solute dielectric constant (pdie) was set as 4.

## 3. Results and Discussions

### 3.1. Fluorescence Spectroscopy

Figure 1 shows the effect in the NBD Trp90 fluorescence caused by the addition of piperine in the solution. According to the spectra, there are two fluorescent bands centered at 330 nm and at 485 nm. The first one refers to protein Trp90 fluorescence emission while the second one refers to piperine fluorescence emission. The full-width half maximum (FWHM) for the band at 330 is ±30 nm and for the band at 480 is ±46 nm, which guarantees that the bands do not overlap and allows the fluorescence intensity at 330 nm to be handled accurately. Figure 1 also shows the fluorescence intensity of tryptophan decreased while piperine was added to the solution, which evidenced that Trp90 was quenched. Another characteristic observed is that the Trp90 fluorescence band remained centered at 330 nm during the piperine titration, which showed the fluorophore was not exposed to an environment with a different polarity [31]. 

There are two possible different quenching mechanisms. One is dynamic quenching, when the ligand deactivates the excited form of the protein fluorophore by collisions. Another is static quenching, when there is a complex formation between the protein and the ligand. A simple way to distinguish the quenching mechanisms is by analyzing the Stern-Volmer constants (K_SV_) at different temperatures [14] obtained by Equation (5). If the K_SV_ decreases with the rise in temperature, it is evidence of static quenching. On the other hand, if the K_SV_ increases with the rise in temperature, the quenching mechanism is not directly determined because the increase of K_SV_ may be an effect of either a complex formation drove by entropic factors or an effect of collisions.

A complementary method to determine the quenching mechanism is the association of the steady-state and time-resolved fluorescence data [32]. If the fluorophore is quenched by collisions, the ratio of fluorescence intensities F_0_/F is equivalent to the ratio of fluorophore lifetime τ_0_/τ, e.g.,: F_0_/F = τ_0_/τ. On the other hand, if such equivalence is not verified, static quenching is occurring. In addition, the combination of steady-state and time-resolved fluorescence data can result in a constant known as bimolecular quenching rate constant (k_q_), which can be obtained through Equation (5). This constant is related to processes of diffusion, and in the case that the system is under collisions between the fluorophore and the ligand, the constant cannot exceed the order of 10^10^ M^−1^·s^−1^ [32]; otherwise the quenching is static.

(5) $$\frac{F_{0}}{F}=1+K_{\mathrm{SV}}\cdot \left[\mathrm{piperine}\right]=1+{\;k}_{q}\cdot \tau_{0}\cdot \left[\mathrm{piperine}\right]\;$$

**Figure 2:**
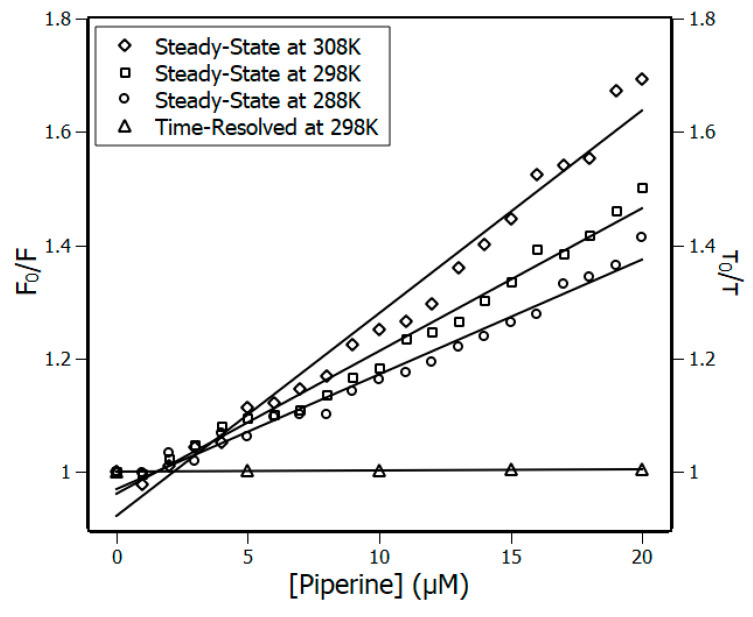
Left ordinate Stern–Volmer plots at three temperatures, 288 K, 298 K and 308 K, and right ordinate time-resolved fluorescence lifetime plot at 298 K; (NBD) = 4 μM, (piperine) = 0–20 μM. R^2^ > 0.98.

The Stern-Volmer plots (Figure 2) exhibited a linear response under piperine titration, indicating a single class of fluorophore in the protein and therefore the presence of one quenching mechanism process [33]. According to the results obtained for the K_SV_ constants presented in Table 1, the increase in temperature also caused the values of the constants to follow it. Figure 2 also shows that piperine poorly affected the Trp90 lifetime, once τ_0_/τ remained close to the unity. Further, according to the plots, no equivalence is found between the ratios of fluorescence intensities and the lifetime values (F_0_/F ≠ τ_0_/τ). The set of these results indicated that the system is under static quenching. To reinforce this indication, an analysis of the bimolecular constant at different temperatures was carried out (Table 1). It was found that for the three temperatures, k_q_ magnitude was of the order of 10^12^ M^−1^·s^−1^, which is two orders of magnitude greater than that observed for collisional quenching (10^10^ M^−1^·s^−1^). In conclusion, all these results revealed that the quenching mechanism is undoubtedly static and therefore a complex is formed by NBD and piperine.

**Figure 3:**
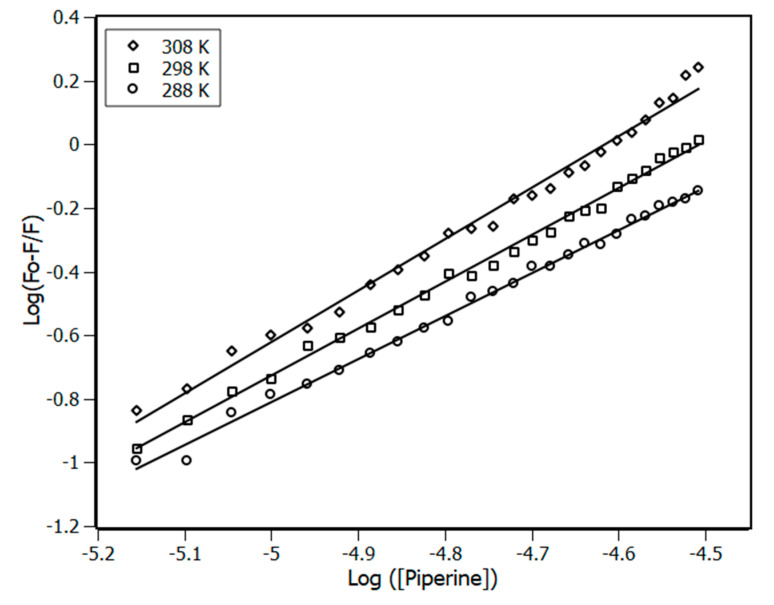
Double-log plots for the fluorescence quenching of NBD (4 μM) in the presence of piperine at 288 K, 298 K and 308 K. R^2^ > 0.99.

Once the complex formation is proven, the next step was to obtain the binding constant (K_a_), applying the binding equilibrium model. The K_a_ was obtained from the plot of Figure 3 using the double-logarithm equation (Equation (6)), which relates the quenching fluorescence intensities with the total concentration of piperine.

(6) $$\log\left(\frac{F_{0}-F}{F}\right)=\log K_{a}-n\cdot \log\left[\mathrm{piperine}\right]\;$$

The results of K_a_ at different temperatures for the first order model (*n* ≈ 1) are presented in Table 1. The binding constants found at different temperatures have a magnitude order of 10^5^ M^−1^, however their values differ at each temperature, meaning that it is under the direct influence of the available thermal energy.

### 3.2. Thermodynamic Parameters

**Figure 4:**
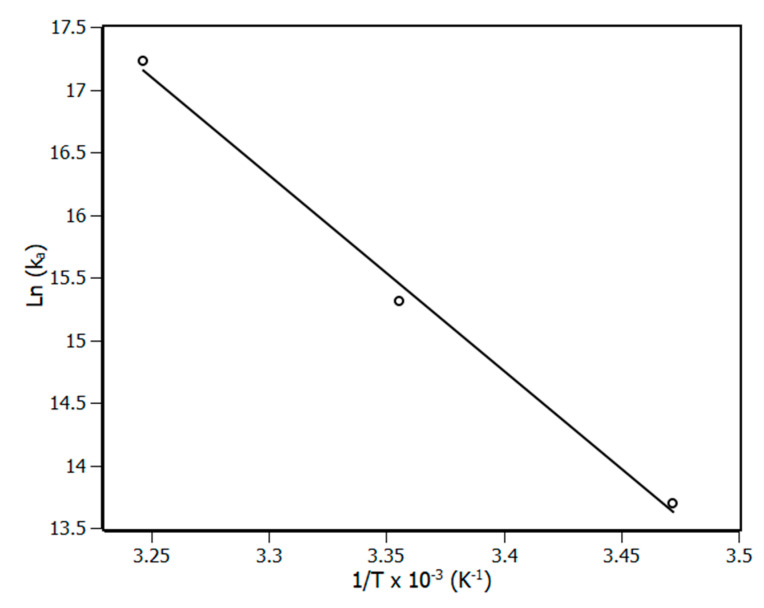
van’t Hoff plot for the complex NBD-piperine at 288 K, 298 K and 308 K. R^2^ > 0.99.

Based on thermodynamic parameters such as ∆S (entropy variation), ∆H (enthalpy variation) and ∆G (Gibbs free variation) it is possible to gain more information about the complex formation and the forces that drive the process [34]. The parameters ∆S and ∆H are obtained from the van’t Hoff plot (Figure 4) according to Equation (7) and ∆G is calculated from Equation (8).

(7) $$\ln K_{a}=-\frac{\Delta H}{R\cdot T}+\frac{\Delta S}{R}\;$$

(8) ΔG=ΔH−TΔS

The results of ∆S, ∆H and ∆G are shown in Table 2. Regarding the results, ∆G values were negative at the range of the applied temperatures, indicating that the complexation was a spontaneous process. Furthermore, the values of ∆G moved to more negative values with the rise in temperature due to the influence of the entropic factor, which favored the complex formation. Moreover, both terms T.∆S and ∆H are positive, which indicated the non-specific interactions as the main contributor for the complexation. In addition, the entropic term is higher than the enthalpic, which reinforced the non-specific characteristic of the interactions.

### 3.3. Interaction Density Function (IDF)

**Figure 5:**
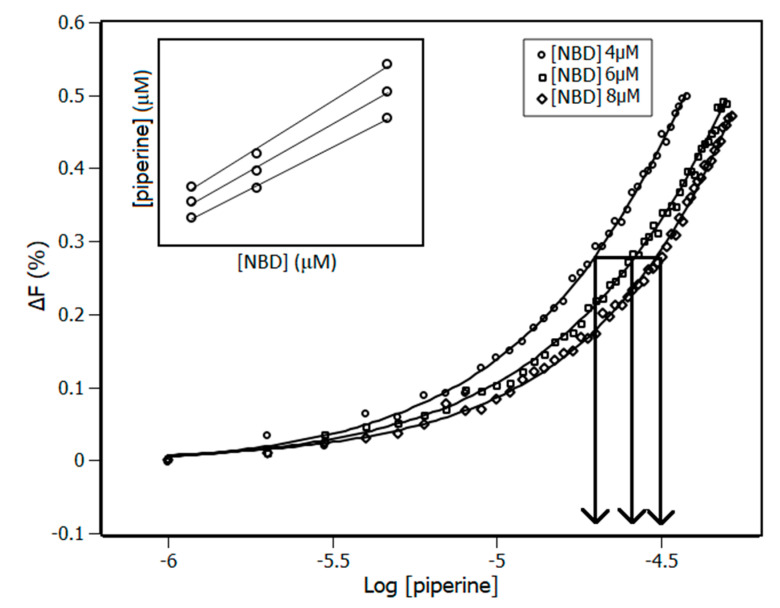
Plot of ∆F versus the log (piperine) obtained from piperine titration experiments with NBD concentrations of 4 μM, 6 μM and 8 μM at 288 K. The inset contains 3 datasets of (piperine) and (NBD) as an example of using Equation (5) to obtain the values of Σν_i_ and (piperine)_free_.

Considering the need to understand the way in which an NBD domain accommodates piperine in its sites, the IDF method was applied to the system as an alternative method in comparison to the binding equilibrium model in order to obtain a more complete description of the system. Interaction density function is a methodology used to treat experimental data, but it is different from the binding equilibrium model, as IDF does not make use of any model a priori and it is based on mass conservation law [35]. The advantage of applying IDF is the possibility of determining the real number of binding sites and identifying any matching factor between the sites. IDF considers that, if the free ligand concentration ((piperine)_free_) is the same for two or more solutions at different concentrations of total protein ((NBD)), the average interaction density (Συ_i_) will also be the same and consequently the system will have the same variation on the percentage of quenching (ΔF). The percentage of fluorescence quenching is given by Equation (9), where F and F_0_ are the observed fluorescence signal with and without piperine, respectively. Figure 5 shows the plot of ΔF against the log [piperine] for three concentrations of NBD adjusted by a sigmoidal function.

(9) $$\Delta F=\frac{\left|F-F_{0}\right|}{F_{0}}\;\cdot \;100\%\;$$

Free ligand concentration and the average interaction density are related to each other through the expression of mass conservation (Equation (10)).

(10) $$\left[\mathrm{piperine}\right]={\left[\mathrm{piperine}\right]}_{\mathrm{free}}+\;\left(\sum^{\;}\nu_{i}\right)\cdot \left[\mathrm{NBD}\right]$$

By means of the plot shown in Figure 5, the values of (NBD) and (piperine) for each ΔF was obtained. The inset of Figure 5 shows an example of 3 datasets of the plot of (piperine) versus (NBD) for each ΔF, in which Σν_i_ is obtained from the slope, and (piperine)_free_ is obtained from the y-intercept of the linear function.

**Figure 6:**
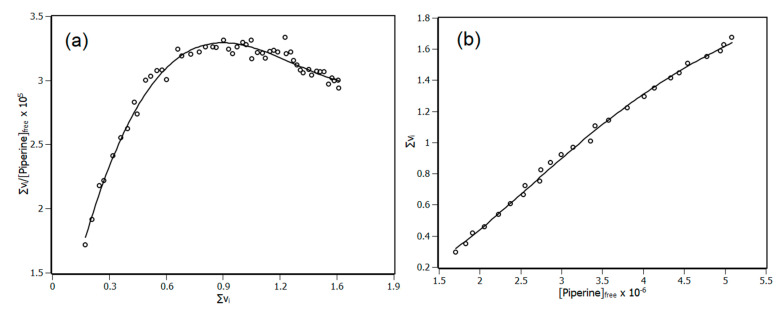
(**a**) Scatchard plot for the interaction of NBD and piperine obtained at 288 K based on interaction density function (IDF) data. (**b**) Hill plot for the interaction of NBD and piperine obtained at 288 K based on IDF data. R^2^ > 0.99.

With the parameters Σν_i_ and (piperine)_free_ obtained from IDF, a Scatchard plot was built (Figure 6a), an important source of information about cooperativity. It may reveal if the binding sites of the protein are equivalents or non-equivalents, if there is cooperativity among them as well as if the cooperativity is positive or negative [36,37]. Figure 6a shows a line profile with negative concavity, pointing out positive cooperativity. Although Scatchard’s plot reveals the occurrence of cooperativity, it does not identify the number of sites that NBD has. To complement this information, it is necessary to use the Hill’s plot to disclose additional features such as the number of binding sites (*n*) and the binding constants (K_b_) [38,39]. The Hill’s plot is shown in Figure 6b, whose parameters n and K_b_ were obtained by Equation (11). The parameter h is called the Hill coefficient, which indicates the type of cooperativity. The Hill coefficient can assume values of >1, <1 and =1 indicating positive cooperativity, negative cooperativity and non-cooperativity, respectively.

(11) $$\sum^{\;}\nu_{i}=\underset{j}{\sum }\frac{n.{\left(k_{b}\cdot \left[\mathrm{piperine}{]}_{\mathrm{free}}\right]\right)}^{h}}{1+\;{\left(k_{b}\cdot \left[\mathrm{piperine}{]}_{\mathrm{free}}\right]\right)}^{h}}\;\;$$

Regarding the results obtained by the Hill equation, the protein has two equivalent binding sites with K_b_ = (2.67 ± 0.12) × 10^5^ M^−1^ and positive cooperativity (h = 2.4). The results show that both methods (Scatchard and Hill) are in full agreement, revealing a positive cooperativity system. Further, the binding constant found with Hill’s method is in the magnitude order of 10^5^ M^−1^, which is in agreement with that found by the binding equilibrium method. Both methods (the binding equilibrium and Hill methods) differed somewhat in terms of the absolute value of the binding constant. This difference found is due to the fact that the binding equilibrium method uses a first order chemical reaction model while the Hill method does not, as discussed previously in the literature [16,31,40].

### 3.4. Circular Dichroism

**Figure 7:**
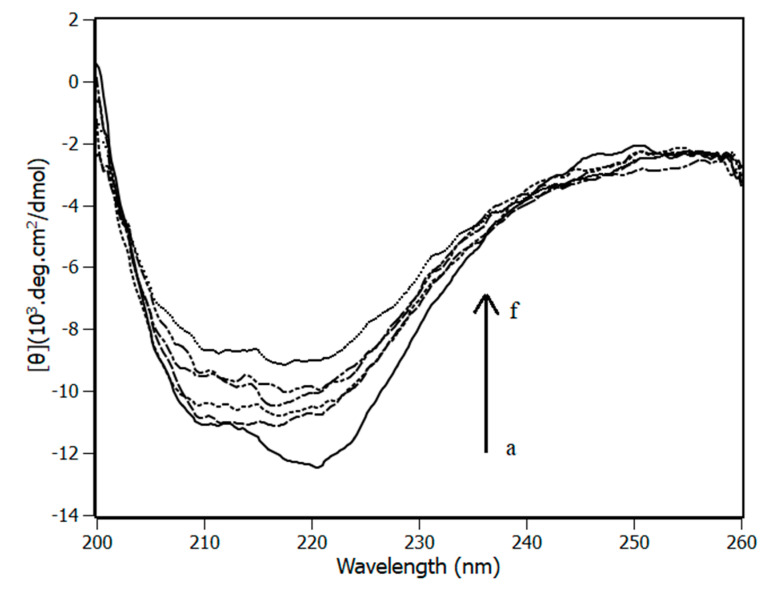
NBD circular dichroism experiments at 298 K in the presence and absence of piperine. Aliquots of piperine were added to the solution from a → f = 1:0 → 1:12.5.

A protein that presents cooperativity when interacting with a ligand may be susceptible to conformational changes, adjusting its structure. To have a complete description about the influence of piperine in NBD structure, circular dichroism experiments were performed (Figure 7).

The circular dichroism spectrum of NBD in solution has well-defined bands at 208 nm and 222 nm, which is characteristic of an alpha-helices secondary structure. In order to obtain more details about the secondary structures, the protein spectra were deconvolved by CDPro software aided by the CONTINNL algorithm with 43 soluble proteins spectra deposited in its library. According to the analyses, NBD has 34% of alpha-helices, 16% of beta sheet, 20% of turns and 30% of coil. These results are in good agreement with the data reported in the literature for the same protein using CDNN software [41].

According to the results presented in Figure 7, the protein underwent structural changes during the titration. At the highest concentration of piperine and NDB (1:12.5), its secondary structure was composed 25% of alpha-helices, 21% of beta-sheet, 22% of turn and 32% of coil. Turn and coil content results did not undergo significant secondary structural changes (≈2%). In the meantime, alpha-helices dropped from 34% to 26% and beta sheet content rose from 16% to 31%. These results revealed that the positive cooperativity found by the Scatchard and Hill methods are followed by the NBD structural changes.

### 3.5. Molecular Modeling

#### 3.5.1. Molecular Docking

**Figure 8:**
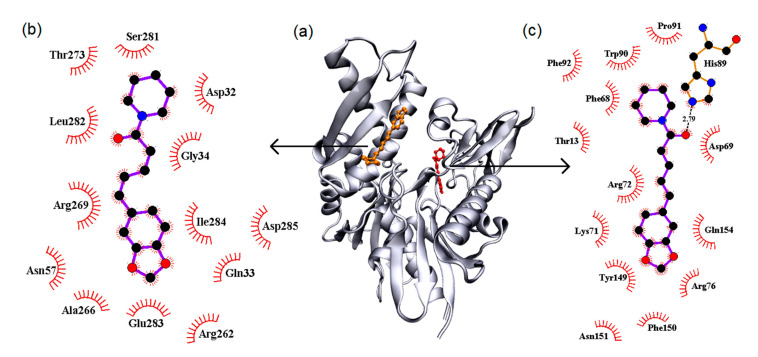
The central picture represents the molecular docking (**a**) in a general view. In red is piperine in binding site 1, in orange in site 2. Pose (**b**) binding environment of site 2, and (**c**) site 1 with a dotted line indicating the hydrogen bonds.

Molecular docking was applied to predict the two binding sites found experimentally. The binding environment of the two sites is shown in Figure 8 and according to the results, the predominant molecular interactions are non-specific, with just one hydrogen bond with 2.79 Å of length performed by piperine and His89. This result is in agreement with the experimental van’t Hoff analysis that indicated the non-specific interactions were predominant. The binding site 1 is composed of the non-polar amino acids Pro91, Phe68, Phe92, Phe150 and Trp90, by the polar amino acids Gln154, Asn151, Tyr149, Thr13 and His89, by amino acids charged positively, Arg76, Lys71, Arg72 and His89, and a negative Asp69. 

Binding site 2 is composed of the non-polar amino acids Gly34, Ile284, Ala266 and Leu282, by the polar amino acids Ser281, Gln33, Asn57 and Thr273, by amino acids charged positively, Arg262 and Arg269, and by amino acids charged negatively, Asp32, Asp285 and Glu283.

#### 3.5.2. Molecular Dynamics

**Figure 9:**
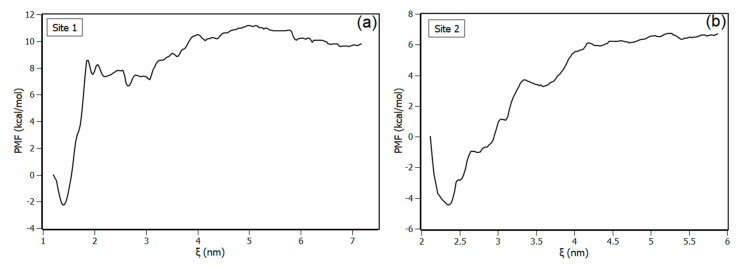
Potential of mean force (PMF) for the dissociation of piperine from NBD, (**a**) for binding site 1 and for (**b**) binding site 2.

The two most promising binding sites predicted by molecular docking were explored by the umbrella sampling method. Figure 9 shows the potential of mean force profiles resulting from the complex dissociation obtained for the two binding sites. According to the results, both binding site profiles presented the minimum of energy at the configuration predicted by molecular docking, which indicated that the two configurations are stable. Potential of mean force (PMF) profiles also revealed that the energetic barrier to unbind piperine from the binding sites is higher in site 1 than in site 2. The standard free energy for the binding sites (∆G_pred_) was determined from WHAM analyses, with (−45 ± 5) kJ/mol for binding site 1 and (−41.8 ± 4) kJ/mol for binding site 2. Although the PMF profiles revealed that the binding sites are distinct in terms of the energetic barrier profile to dissociate piperine, the binding free energy values determined with WHAM analyses are very close considering the statistical errors, corroborating the results obtained from Hill’s plot that showed equivalence between the binding affinities of both sites.

The lower computational time consuming method MM/PBSA was employed to highlight the effect on binding free energy caused by piperine into the two binding sites (Table 3). It is an advantageous method to compare binding energies and comprehend which interaction has the highest binding affinity. The binding free energy calculations were performed in two steps. The first step consisted of calculating the binding free energy of the complex with one binding site occupied. The second step consisted of calculating the binding free energy of the complex with both binding sites occupied. According to the results summarized in Table 3, the binding free energy when both binding sites were accessed (columns 4 and 5) is more negative than when just one site is accessed by piperine (columns 2 and 3), with approximately 10 kJ/mol of difference. These results showed a higher binding affinity of piperine for NBD accessing both sites than a single site, which is evidence of cooperativity, in agreement with the experimental fluorescence analyses of the interaction.

The root mean square fluctuation (RMSF) of NBD simulated in the absence of ligand disclosed the dynamic of the protein residues, indicating that NBD presented some specific regions with high fluctuation (>0.25 nm). The RMSF of NBD simulated with site 1 and 2 occupied by piperine showed that the interaction with ligand induced a decrease in the fluctuation of some regions that was previously high; such regions where marked with blue dots in Figure S7a. According to the results, most of the amino acids that exhibited expressive changes in RMSF surround piperine in binding site 2 (blue new cartoon regions of Figure S7b,c), while it was noted that there were few fluctuation changes for residues surrounding piperine in binding site 1.

## 4. Conclusions

In the present work, the interaction between piperine and NBD was investigated by means of experimental and computational molecular biophysical tools. Steady-state and time-resolved fluorescence showed that NBD in the presence of piperine presented a static-quenching process, which means that a complex was formed. Fluorescence spectroscopy revealed with a Scatchard plot and Hill’s method an important feature about the interaction of NBD and piperine, which was the presence of positive cooperativity between the two binding sites. In addition, the thermodynamic parameters were disclosed, showing the spontaneity of a complex formation (ΔG < 0 kJ/mol) for the three temperatures. Circular dichroism revealed that the protein underwent conformational changes due to the interaction with piperine. The binding sites were unveiled by molecular docking and molecular dynamics, which reinforced the binding free energy found experimentally. Molecular dynamics along with MM/PBSA reinforced the positive cooperativity found experimentally, showing that the binding free energy was more negative when both binding sites were occupied by piperine. In other words, the affinity was higher under this condition than when just one binding site was occupied. A multispectroscopic evaluation aided by molecular docking and dynamics elucidated in detail the NBD/piperine molecular interaction, which may support further drug discovery studies.

## Figures and Tables

**Table 1:** Stern-Volmer constant (K_SV_), bimolecular constants (k_q_) and binding constant (K_a_) for the complex NBD and piperine at 288, 298 and 308 K.

Temperature (K)	Stern-Volmer (K_SV_) ×10^4^ M^−1^	Bimolecular (K_q_) ×10^12^ M^−1^·s^−1^	Binding (K_a_) ×10^5^ M^−1^
288	2.02 ± 0.07	9.18 ± 0.01	8.82 ± 0.07
298	2.52 ± 0.08	11.45 ± 0.01	44.60 ± 0.10
308	3.58 ± 0.1	16.27 ± 0.01	301.37 ± 1.1

**Table 2:** Thermodynamic parameters of the complex NBD-piperine at temperatures of 288 K, 298 K and 308 K.

T (K)	∆G (kJ/mol)	∆H (kJ/mol)	T.∆S (kJ/mol)
288	−32.65 ± 1.31	130.13 ± 8.69	162.78 ± 8.4
298	−38.3 ± 1.94	130.13 ± 8.69	168.43 ± 8.69
308	−43.95 ± 2.06	130.13 ± 8.69	174.01 ± 8.98

**Table 3:** Energies obtained from molecular mechanics Poisson Boltzmann surface area (MM/PBSA) for piperine occupying both sites and for piperine occupying just one site. Van der Waals, Electrostatic, polar solvation and SASA were obtained from a PBSA calculation.

Energies (kJ/mol)	Site 1	Site 2	Both Sites Occupied	
			Site 1	Site 2
Binding free energy ∆G	−35.22 ± 2.45	−34.59 ± 2.60	−49.92 ± 3.01	−46.15 ± 2.03
van der Waals	−97.63 ± 1.86	−119.61 ± 2.25	−73.74 ± 1.98	−96.12 ± 1.45
Electrostatic	−11.76 ± 0.85	−25.23 ± 0.95	−2.80 ± 1.73	−12.31 ± 1.17
Polar solvation	86.80 ± 2.89	124.32 ± 3.52	36.25 ± 3.34	73.88 ± 2.1
SASA	−12.64 ± 0.24	−14.07 ± 0.23	−9.64 ± 0.25	−11.59 ± 0.12

## Supplementary Materials

The following are available online at https://www.mdpi.com/2227-9059/8/12/629/s1. Figure S1: Time-resolved fluorescence decay of (a) NBD with Piperine (→e) from 0 to 20 µM. [IL-1β] = 10 μM, T = 298 K and λex = 295 nm, Figure S2: Molecular docking clusters with their respectives energy scores, Figure S3: The atoms picked to define the reaction coordinate (ξ) for (a) binding site 1 and (b) binding site 2, Figure S4: Pulling profile during the pulling simulation. Y-axis is the value of reaction coordinate (ξ) and x-axis is the time of simulation, Figure S5: Configuration histograms of the pulling in z-axis with the windows distance as being 0.1 nm for (a) binding site 1 and (b) binding site 2, Figure S6: Root mean square deviation (RMSD) of (a) NBD in the presence of piperine and (b) piperine occupying the binding sites, Figure S7: (a) Root mean square fluctuation (RMSF) of NBD residues in the absence and presence of piperine in both binding sites (red and black, respectively), blue dots represent the residues that altered the fluctuation from high to low when the ligands were inserted in the binding sites. (b) and (c) Blue cartoon represents the regions that altered the fluctuation from high to low when the ligands were inserted in the binding sites. Red and Orange represent piperine in site 1 and in site 2, respectively, Table S1: Tryptophan lifetime in different stoichiometries NBD: Piperine.

## References

[1] Dubrez L., Causse S., Bonan N.B., Dumetier B., Garrido C. (2019). Heat-shock proteins: Chaperoning DNA repair. Oncogene.

[2] Jones A.M., Westwood I.M., Osborne J.D., Matthews T.P., Cheeseman M.D., Rowlands M.G., Jeganathan F., Burke R., Lee D., Kadi N. (2016). A fragment-based approach applied to a highly flexible target: Insights and challenges towards the inhibition of HSP70 isoforms. Sci. Rep..

[3] Asea A., Kraeft S.-K., Kurt-Jones E.A., Stevenson M.A., Chen L.B., Finberg R.W., Koo G.C., Calderwood S.K. (2000). HSP70 stimulates cytokine production through a CD14-dependant pathway, demonstrating its dual role as a chaperone and cytokine. Nat. Med..

[4] Asea A., Rehli M., Kabingu E., Boch J.A., Baré O., Auron P.E., Stevenson M.A., Calderwood S.K. (2002). Novel signal transduction pathway utilized by extracellular HSP70 role of Toll-like receptor (TLR) 2 and TLR4. J. Biol. Chem..

[5] Somensi N., Brum P.O., de Miranda Ramos V., Gasparotto J., Zanotto-Filho A., Rostirolla D.C., da Silva Morrone M., Moreira J.C.F., Gelain D.P. (2017). Extracellular HSP70 activates ERK1/2, NF-kB and pro-inflammatory gene transcription through binding with RAGE in A549 human lung cancer cells. Cell. Physiol. Biochem..

[6] Pahl H.L. (1999). Activators and target genes of Rel/NF-κB transcription factors. Oncogene.

[7] Evans C.G., Chang L., Gestwicki J.E. (2010). Heat shock protein 70 (hsp70) as an emerging drug target. J. Med. Chem..

[8] de Oliveira A.A., Faustino J., de Lima M.E., Menezes R., Nunes K.P. (2019). Unveiling the interplay between the TLR4/MD2 complex and HSP70 in the human cardiovascular system: A computational approach. Int. J. Mol. Sci..

[9] Cheeseman M.D., Westwood I.M., Barbeau O., Rowlands M., Dobson S., Jones A.M., Jeganathan F., Burke R., Kadi N., Workman P. (2016). Exploiting protein conformational change to optimize adenosine-derived inhibitors of HSP70. J. Med. Chem..

[10] Zazeri G., Povinelli A.P.R., Le Duff C.S., Tang B., Cornelio M.L., Jones A.M. (2020). Synthesis and Spectroscopic Analysis of Piperine-and Piperlongumine-Inspired Natural Product Scaffolds and Their Molecular Docking with IL-1β and NF-κB Proteins. Molecules.

[11] Ying X., Chen X., Cheng S., Shen Y., Peng L., Xu H. (2013). Piperine inhibits IL-β induced expression of inflammatory mediators in human osteoarthritis chondrocyte. Int. Immunopharmacol..

[12] Bang J.S., Choi H.M., Sur B.-J., Lim S.-J., Kim J.Y., Yang H.-I., Yoo M.C., Hahm D.-H., Kim K.S. (2009). Anti-inflammatory and antiarthritic effects of piperine in human interleukin 1β-stimulated fibroblast-like synoviocytes and in rat arthritis models. Arthritis Res. Ther..

[13] Ying X., Yu K., Chen X., Chen H., Hong J., Cheng S., Peng L. (2013). Piperine inhibits LPS induced expression of inflammatory mediators in RAW 264.7 cells. Cell. Immunol..

[14] Lakowicz J.R. (2004). Principles of Fluorescence Spectroscopy.

[15] Sreerama N., Woody R.W. (2000). Estimation of protein secondary structure from circular dichroism spectra: Comparison of CONTIN, SELCON, and CDSSTR methods with an expanded reference set. Anal. Biochem..

[16] Zazeri G., Povinelli A.P.R., de Lima M.F., Cornélio M.L. (2019). Experimental Approaches and Computational Modeling of Rat Serum Albumin and Its Interaction with Piperine. Int. J. Mol. Sci..

[17] Morris G.M., Huey R., Lindstrom W., Sanner M.F., Belew R.K., Goodsell D.S., Olson A.J. (2009). AutoDock4 and AutoDockTools4: Automated docking with selective receptor flexibility. J. Comput. Chem..

[18] Morris G.M., Goodsell D.S., Pique M.E., Lindstrom W., Huey R., Forli S., Hart W.E., Halliday S., Belew R., Olson A.J. (2010). User Guide AutoDock Version 4.2. Automated Docking of Flexible Ligands to Flexible Receptors.

[19] Humphrey W., Dalke A., Schulten K. (1996). VMD: Visual molecular dynamics. J. Mol. Graph..

[20] Wallace A.C., Laskowski R.A., Thornton J.M. (1995). LIGPLOT: A program to generate schematic diagrams of protein-ligand interactions. Protein Eng. Des. Sel..

[21] Oostenbrink C., Villa A., Mark A.E., Van Gunsteren W.F. (2004). A biomolecular force field based on the free enthalpy of hydration and solvation: The GROMOS force-field parameter sets 53A5 and 53A6. J. Comput. Chem..

[22] Van Der Spoel D., Lindahl E., Hess B., Groenhof G., Mark A.E., Berendsen H.J.C. (2005). GROMACS: Fast, flexible, and free. J. Comput. Chem..

[23] Wu Y., Tepper H.L., Voth G.A. (2006). Flexible simple point-charge water model with improved liquid-state properties. J. Chem. Phys..

[24] Bussi G., Donadio D., Parrinello M. (2007). Canonical sampling through velocity rescaling. J. Chem. Phys..

[25] Hess B., Bekker H., Berendsen H.J.C., Fraaije J.G.E.M. (1997). LINCS: A linear constraint solver for molecular simulations. J. Comput. Chem..

[26] Batcho P.F., Case D.A., Schlick T. (2001). Optimized particle-mesh Ewald/multiple-time step integration for molecular dynamics simulations. J. Chem. Phys..

[27] Parrinello M., Rahman A. (1981). Polymorphic transitions in single crystals: A new molecular dynamics method. J. Appl. Phys..

[28] Roux B. (1995). The calculation of the potential of mean force using computer simulations. Comput. Phys. Commun..

[29] Kumar S., Rosenberg J.M., Bouzida D., Swendsen R.H., Kollman P.A. (1992). The weighted histogram analysis method for free-energy calculations on biomolecules. I. The method. J. Comput. Chem..

[30] Kumari R., Kumar R., Consortium O.S.D.D., Lynn A. (2014). g_mmpbsa A GROMACS tool for high-throughput MM-PBSA calculations. J. Chem. Inf. Model..

[31] Povinelli A.P.R., Zazeri G., de Freitas Lima M., Cornélio M.L. (2019). Details of the cooperative binding of piperlongumine with rat serum albumin obtained by spectroscopic and computational analyses. Sci. Rep..

[32] Lakowicz J.R., Weber G. (1973). Quenching of fluorescence by oxygen. Probe for structural fluctuations in macromolecules. Biochemistry.

[33] Soares S., Mateus N., De Freitas V. (2007). Interaction of different polyphenols with bovine serum albumin (BSA) and human salivary α-amylase (HSA) by fluorescence quenching. J. Agric. Food Chem..

[34] Ross P.D., Subramanian S. (1981). Thermodynamics of protein association reactions: Forces contributing to stability. Biochemistry.

[35] Lohman T.M., Bujalowski W. (1991). Thermodynamic methods for model-independent determination of equilibrium binding isotherms for protein-DNA interactions: Spectroscopic approaches to monitor binding. Methods Enzymol..

[36] Scatchard G. (1949). The attractions of proteins for small molecules and ions. Ann. N. Y. Acad. Sci..

[37] Povinelli A.P.R., Zazeri G., Cornélio M.L. (2019). Molecular Mechanism of Flavonoids Using Fluorescence Spectroscopy and Computational Tools. Flavonoids-A Coloring Model For Cheering Up Life.

[38] Bordbar A.K., Saboury A.A., Moosavi-Movahedi A.A. (1996). The shapes of Scatchard plots for systems with two sets of binding sites. Biochem. Educ..

[39] Barcroft J., Hill A.V. (1910). The nature of oxyhaemoglobin, with a note on its molecular weight. J. Physiol..

[40] Zazeri G., Povinelli A.P.R., de Freitas Lima M., Cornélio M.L. (2020). The Cytokine IL-1β and Piperine Complex Surveyed by Experimental and Computational Molecular Biophysics. Biomolecules.

[41] Borges J.C., Ramos C.H.I. (2006). Spectroscopic and thermodynamic measurements of nucleotide-induced changes in the human 70-kDa heat shock cognate protein. Arch. Biochem. Biophys..

