# Supplementary material for: Detailed Characterization of the Cooperative Binding of Piperine with Heat Shock Protein 70 by Molecular Biophysical Approaches
Source: Biomedicines. 2020 Dec 18;8(12):629. doi: 10.3390/biomedicines8120629 (PMC7766160; doi:10.3390/biomedicines8120629)
Supplement: Supplementary file 1 [file biomedicines-08-00629-s001.pdf]

biomedicines

MDPI

# Supplementary material

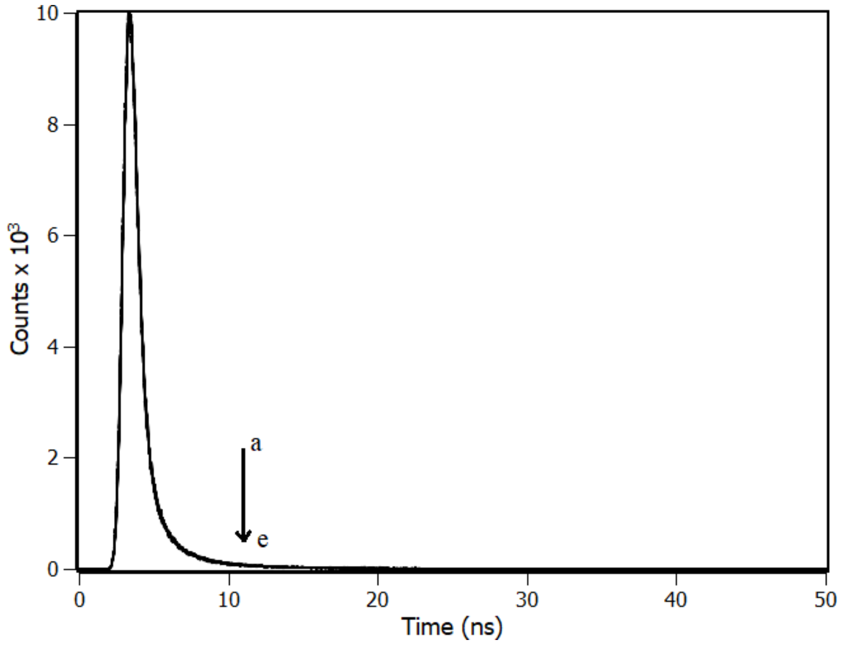


**Figure S1.** Time-resolved fluorescence decay of (a) NBD with Piperine (→e) from 0 to 20μM. [IL-1β] =. 10μM, T = 298K and $\lambda_{ex}$ = 295nm.

**Table S1.** Tryptophan lifetime in different stoichiometries NBD:Piperine.

| [piperine](μM) | α1 | τ1 (ns) | α2 | τ2 (ns) | τavg (ns) |
|---|---|---|---|---|---|
| **0** | 0.78 | 0.22 | 0.73 | 3.31 | 2.19 |
| **5** | 0.774 | 0.23 | 0.72 | 3.29 | 2.19 |
| **10** | 0.78 | 0.22 | 0.73 | 3.32 | 2.19 |
| **15** | 0.79 | 0.20 | 0.75 | 3.41 | 2.18 |
| **20** | 0.79 | 0.21 | 0.74 | 3.37 | 2.18 |

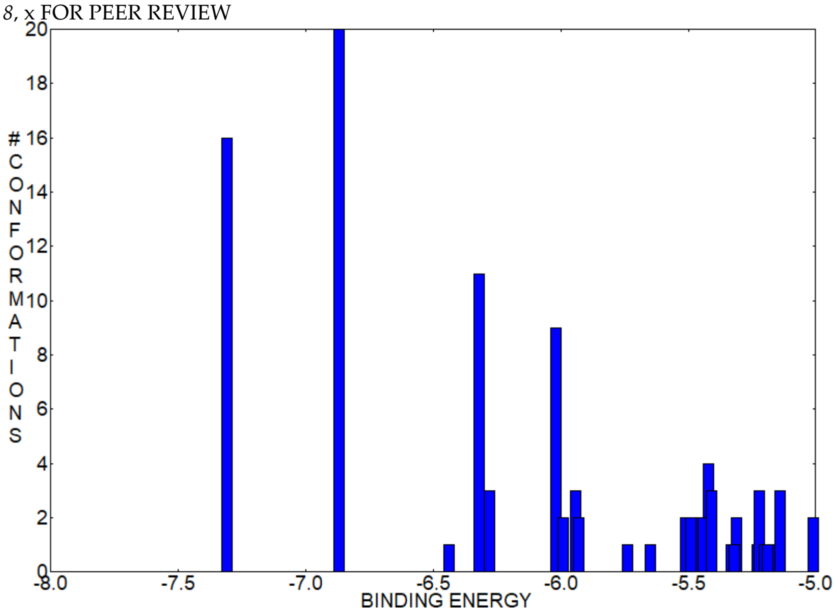


**Figure S2.** Molecular docking clusters with their respectives energy scores.

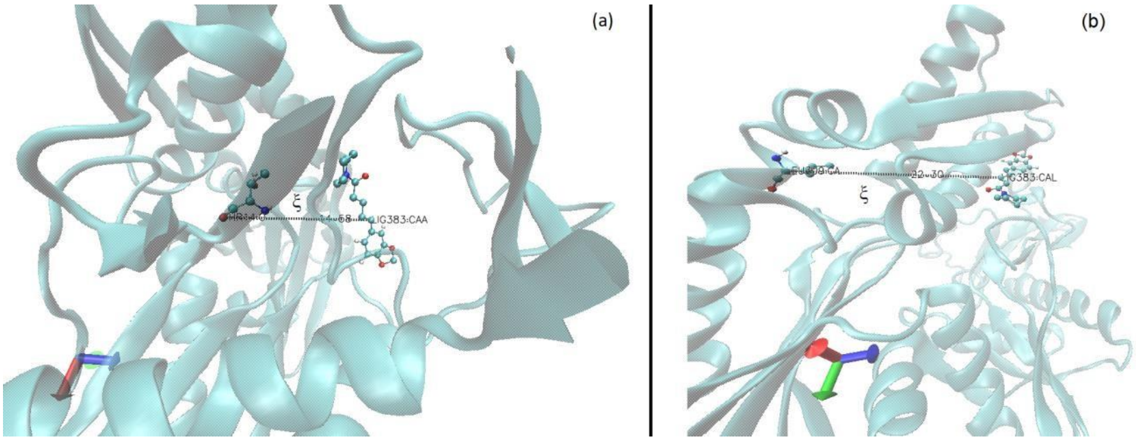


**Figure S3.** The atoms picked to define the reaction coordinate (ξ) for (a) binding site 1 and (b) binding site 2.

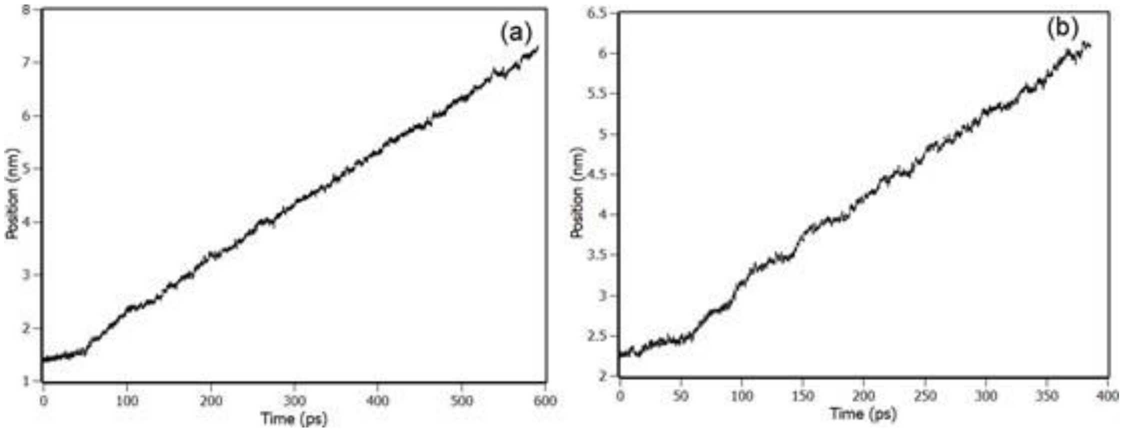


**Figure 4.** Pulling profile during the pulling simulation. Y-axis is the value of reaction coordinate (ξ) and x-axis is the time of simulation.

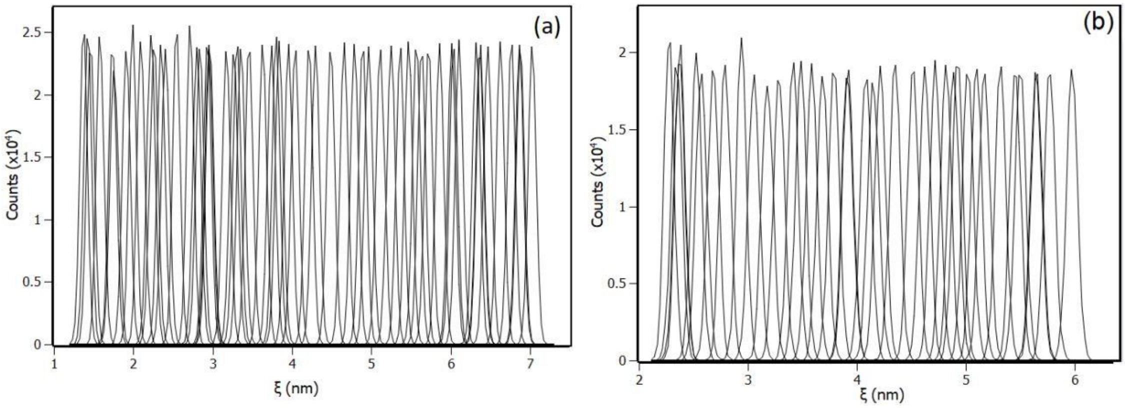


**Figure S5.** Configuration histograms of the pulling in z-axis with the windows distance as being 0.1nm for (a) binding site 1 and (b) binding site 2.

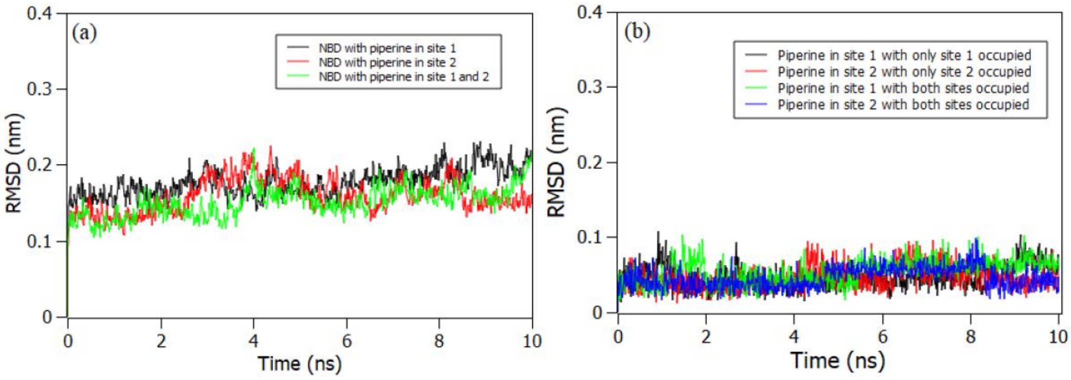


**Figure S6:** Root mean square deviation (RMSD) of (a) NBD in the presence of piperine and (b) piperine occupying the binding sites.

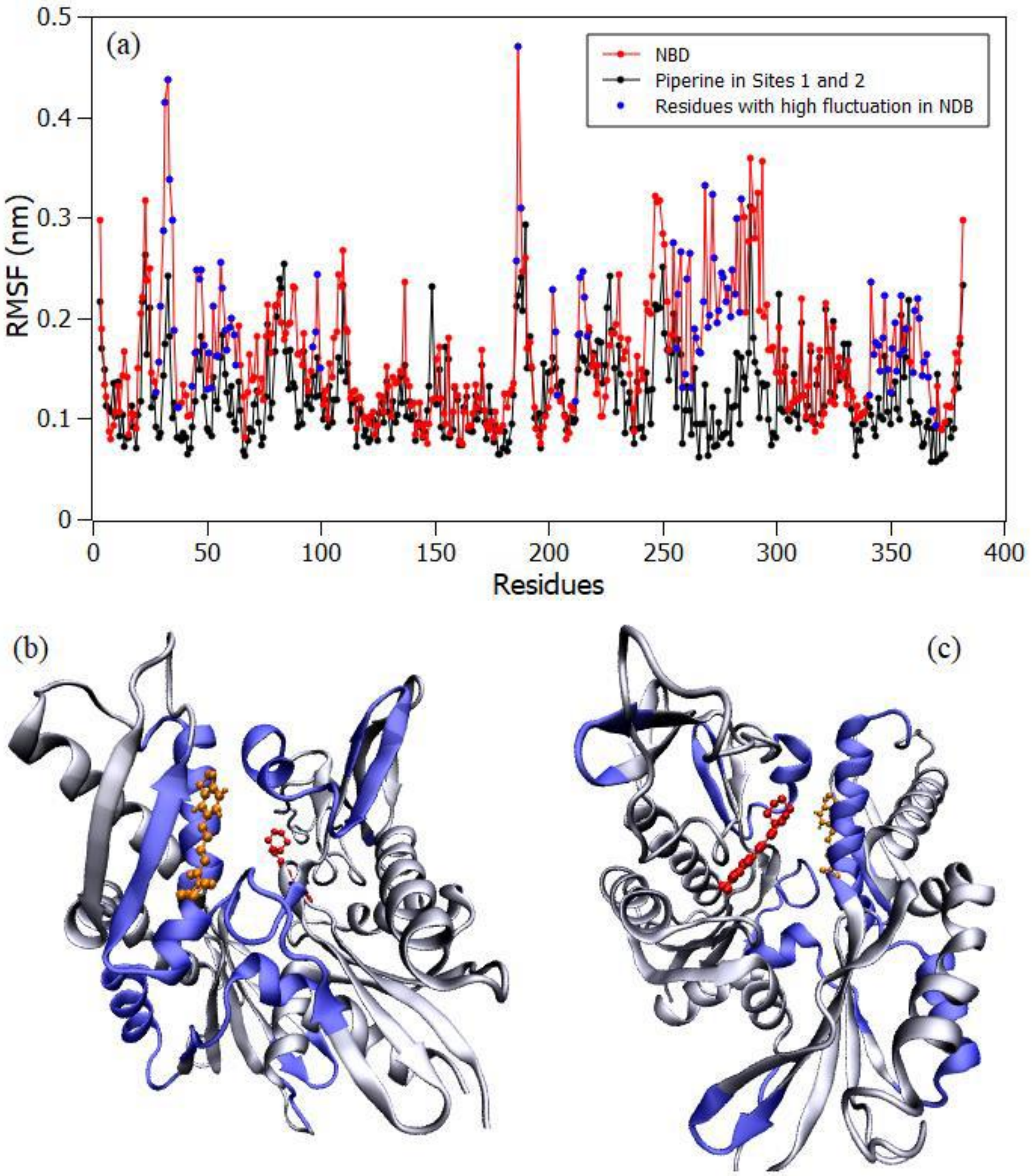


**Figure 7.** (**a**) Root mean square fluctuation (RMSF) of NBD residues in the absence and presence of piperine in both binding sites (red and black, respectively), blue dots represent the residues that altered the fluctuation from high to low when the ligands were inserted in the binding sites. (**b**) and (**c**) Blue cartoon represents the regions that altered the fluctuation from high to low when the ligands were inserted in the binding sites. Red and Orange represent piperine in site 1 and in site 2, respectively.
